# Impact of interventions to reduce sugar-sweetened beverage intake in children and adults: a protocol for a systematic review and meta-analysis

**DOI:** 10.1186/s13643-015-0008-4

**Published:** 2015-02-21

**Authors:** Elisa J Vargas-Garcia, Charlotte EL Evans, Janet E Cade

**Affiliations:** Nutritional Epidemiology Group, School of Food Science and Nutrition, University of Leeds, Woodhouse Lane, Leeds, LS2 9JT UK

**Keywords:** Sugar-sweetened beverage, Behaviour change, Intervention, Systematic review, Meta-analysis, Randomised controlled trial, Public health

## Abstract

**Background:**

Sugar-sweetened beverages (SSBs) have been stressed as relevant targets of public health interventions considering the negative outcomes derived from their excessive intake. Though the evidence from published literature grows to support a cause-and-effect association of SSBs with obesity and other diseases, little is known on the effectiveness that strategies alone or as part of multi-component programmes have had to influence this particular dietary behaviour across all ages. Therefore, this review and meta-analysis aim to evaluate the effect that interventions have had to decrease their consumption or increase water intake in children and adults so as to guide the design of future programmes and inform policy making.

**Methods:**

Included studies in this review will be randomised controlled trials and quasi-experimental interventions (with a control group) that have reported baseline and post-intervention intakes of SSBs or water and that have been published from 1990 in any language. A thorough search will be performed in MEDLINE, EMBASE, Scopus, Web of Science, Cochrane’s central register of controlled trials, and the Global Health Library. Two independent reviewers will conduct initial screening of potentially included articles and will later extract data to analyse domains of intervention design and delivery (with emphasis on behaviour change techniques used as rationale), as well as results in changes on consumption patterns and behavioural determinants. Internal and external validity of each study will also be appraised. A meta-analysis will be performed if a sufficient number of studies are available, and if not, a narrative review will be conducted instead.

**Discussion:**

The results from this review aim to strengthen public health initiatives tackling obesity through improvements in non-alcoholic drinking patterns. As a subject of growing attention globally, this review will help determine which strategies available are the most effective in different contexts. Knowledge gained from this work will also aid resource allocation in future research and government agendas.

**Systematic review registration:**

PROSPERO: CRD42014013436.

**Electronic supplementary material:**

The online version of this article (doi:10.1186/s13643-015-0008-4) contains supplementary material, which is available to authorized users.

## Background

Obesity represents one of the most important public health challenges of the modern era. Projections for 2030 have estimated that up to 2.16 billion and 1.12 billion adults will be overweight and obese, respectively [1]. Yet, it has been recognised that the economic, social and health consequences will be greater for low- and lower-middle-income countries facing nutritional transitions such as those in Northern Africa, the Middle East, Asia and Latin America [2].

Several responses have been undertaken to counteract this problem mainly through interventions that have addressed modifiable factors - such as healthy eating and physical activity [3-6]. Results nevertheless have been inconsistent in the long term, partly due to a lack of commitment and allocated resources from national levels for evaluation and to guarantee their sustainability [7].

In the majority of obesity prevention programmes, strategies have focused on discouraging high intakes of fat (mainly saturated and *trans*) and added sugars in food and beverages. Evidence has additionally supported the potential to target individual dietary elements that contribute to higher energy intakes and that increase the risk of developing obesity [8,9]. This is the case with sugar-sweetened beverages (SSBs), which are high sources of energy with poor nutritional and satiating values [9,10]. SSBs are made up of naturally occurring caloric sweeteners such as sucrose (50% glucose and 50% fructose), fruit juice concentrates or more frequently high fructose corn syrup (45% glucose and 55% fructose) [11]. The latter, in particular, has been attributed as one of the main contributors to the adverse health effects from SSBs due to the metabolic pathways of fructose degradation (exacerbating triglyceride synthesis, insulin resistance and uric acid production) [12]. However, as the use of any caloric sweetener in beverages appears to have similar acute responses in the body, more robust study designs and data are warranted to determine detrimental health outcomes in the longer term [13-17].

Soft drinks are the leading source of added sugars in the American diet, accounting for 9.5% of total energy intake in adolescents and young adults and up to 6.2% in children from 6 to 11 years old [18]. Data from consumption patterns in the United Kingdom (UK) indicates that non-alcoholic beverages contribute to approximately 6% of daily total energy intake in boys and girls (4 to 18 years old), 4% in men and women (19 to 64 years old) and 2% in older adults [19]. On the other hand, soft drinks (carbonated and noncarbonated beverages containing sugar as well as fruit drinks) account for approximately 10% of total daily energy intake in Mexican children and adults [20-22]. A similar trend of consumption has been also reported for Taiwan, although the most popular sweetened drinks in that country include ice-based beverages and processed juice [23]. Figures on purchasing trends in China and Brazil have shown a steep rise in SSB sales per capita of 269% and 147%, respectively, for The Coca Cola Company over the last decade [24]. Brazil household surveys have identified that 2.1% of total calories consumed come from soft drinks exclusively, whereas in Chile, SSBs are amongst the three main products of food expenditure within the poorest sectors of society [25,26]. The former highlights that sugar-sweetened beverage intake is not a problem exclusively of the developed world but also one affecting developing countries [1]. Indeed, it has been emphasised that it is those on a lower income that have higher odds of engaging in unhealthier dietary patterns such as drinking SSBs - since access and availability to these is not limited to the least deprived [27].

Considering the burden of disease derived by obesity and the financial constraints posed to healthcare systems globally, policy makers and governments around the world have widely supported and joined efforts in improving low- or non-caloric beverage consumption patterns. Actions taken have encompassed interventions to decrease consumption of SSBs or/and increase water intake at community levels, through school policies and media coverage (health campaigns). Political measures like taxation and marketing restrictions have also been implemented.

Nevertheless, there is general recognition about the need for sufficient evidence to help decide the best public health action to decrease sugar-sweetened beverage consumption within populations [9,28]. Though literature has particularly highlighted the importance to address behaviour change in interventions so that effective and successful practice can be achieved both in clinical and public health sectors [29], to-date, there are no reviews available that have evaluated the content of interventions seeking to modify behaviour of SSB consumption. The reviews that are available have focused on the cause-and-effect association of SSB with obesity and other health outcomes and have advocated the need for successful initiatives to promote a change in SSB consumption [16,20,30-33].

As an issue of growing interest internationally, it is then necessary to inform intervention designers as well as higher levels of authority of the interventions that have most success in reducing SSB intake, in order to improve dietary guidelines and health outcomes and ensure better allocation of health resources.

### Aims

The main purpose of this systematic review and meta-analysis is to evaluate the effect of public health interventions to reduce sugar-sweetened beverage intake or increase water intake in children and adults.

Primary objectives include

- ▪ Evaluation of intervention elements or factors generating a change in SSB behaviour (either on their frequency of consumption or amount consumed) in children and adults.
- ▪ Evaluation of intervention elements or factors generating a change in water intake in children and adults.

Secondary objectives

- ▪ Identification and evaluation of the most effective behavioural change techniques targeting SSBs or water intake.
- ▪ Evaluation of programmes' delivery processes and their contribution to achieving sustainable outcomes.
- ▪ Identification of the effectiveness of interventions targeting SSBs or water intake to decrease health inequalities.

## Methods

### How the intervention might work

**Figure 1:**
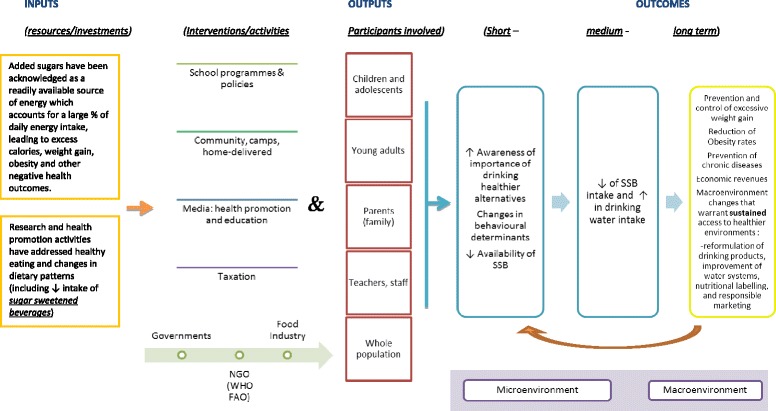
**Decreasing SSB and/or increasing water intake.** Interventions and activities for decreasing SSB and/or increasing water intake in children and adults. SSB, sugar-sweetened beverage.

As portrayed in Figure 1, evidence surrounding the deleterious effects from increased intake of added sugars in the diet (such as those coming from SSBs) has encouraged different initiatives involving a wide range of stakeholders (from children to governmental authorities). By addressing SSB consumption, it is firstly desired to have an impact on participants’ awareness, knowledge and beliefs that could increase their motivation to change this dietary behaviour [34]. By successfully turning attempts at change into action, weight gain can be prevented, and further benefits on a larger scale can be achieved. It should be noted that the macro-environment or context *per se* may stand as a barrier at primary stages of interventions for enabling the development of desirable skills and behaviours particularly in those from disadvantaged backgrounds [29,34].

Research questions

- Can public health interventions reduce the intake of sugar-sweetened beverages in children and adults?
- Can public health interventions increase water intake in children and adults?
- Which intervention components/elements are contributing to reducing SSB intake or increasing water intake in children and adults?
- Which intervention components/elements are hindering or benefiting the implementation/delivery process of programmes targeting consumption of drinking water or SSB?
- Which are the most potentially effective behavioural techniques underpinning changes in SSB or water intake?
- What has been the impact of public health interventions targeting either a reduction in SSB consumption or increase in water intake on reducing health inequalities?

### Description of the Condition/issue

#### What is a sugar-sweetened beverage?

The range of products that fall into such a category is broad as its characterisation and availability varies from country to country. Language discrepancies also pose a difficulty. The lack of a standard definition across different studies has been previously highlighted [35]. Yet, major presence of added sugars is the key element to judge or rate overall healthiness and their inclusion under such a term.

#### Operational definitions

Existing information mainly describes a sugar-sweetened beverage as a non-diet, non-alcoholic and non-dairy cold or warm drink (carbonated or still), with added sugars (derived from energy-yielding sweeteners/sources both natural and processed), including fruit drinks, nectars and cordials with less than 100% fruit juice as well as sports or energy drinks, ready-to-drink sweetened tea and ready-to-drink sweetened coffee [27,36].

In the case of drinking water, this will be considered as water that is intended to be ingested or for human consumption. Other terms frequently found in the literature are potable water, plain water or bottled water.

### Description of the intervention

‘Intervention’ and ‘programme’ will be interchangeable terms used throughout this review. Public health intervention will be understood as a set of actions (within policy, regulatory initiatives, single strategy projects or multi-component programmes) with a coherent objective to bring about change or produce identifiable outcomes seeking to promote health or prevent disease in communities or populations [37].

#### Type of studies

This review will include

- Randomised controlled trials (RCTs) reporting a change in sugar-sweetened beverage or water intake throughout the day as part of the intervention targets (even if they were not explicitly designed to address SSB or drinking water alone).
- Quasi-experimental studies and pilot trials (considering the probable lack of RCTs available).
- Studies that have been published from January 1990 in any language.

This review will exclude

- Observational and small studies (for example, enrolling fewer than ten people in each arm) [38].
- Studies looking at health outcomes (type 2 diabetes, cardiovascular disease and so on) as the primary outcome with no measure of intake of SSB or drinking water being reported.
- Studies addressing gestational diabetes - if no randomisation took place.
- Studies that do not provide an objective measure of SSB (describing frequency of consumption) derived from a standard assessment method (24-h recall, weighed or un-weighed food diary or food frequency questionnaire).
- Studies that do not have baseline and post-intervention information on either primary outcomes (SSB or water consumption).
- Studies that do not have a control group or that do not report any measure of variation such as standard deviation, standard error or 95% confidence intervals will not be included in the meta-analysis but may be considered for the review.
- Alcohol interventions (those targeting reduction of alcohol intake).
- Studies addressing sanitation or hygiene aspects.
- Rehydration and subjective appetite trials (those looking at intake of SSB and acute changes in hormonal or appetite intake).

For the purpose of the present review, the *control group* will also be referred to as the *comparison group* which should be understood as the arm of a programme that did not receive the planned or active intervention (either no activity was given, a ‘usual care’ approach was taken or an alternative intervention was provided).

#### Type of participants

Participants to be included are subjects aged 3 years and over. For mixed-aged groups, only studies where more than 50% of the participants were 3 years or over will be included.

For interventions targeting individuals with morbid obesity (body mass index ≥40 kg/m^2^), metabolic syndrome and chronic diseases such as type 2 diabetes, they will only be reviewed if they were part of a RCT; as by already having a clinical condition or disease, randomisation would diminish or prevent a more favourable prognosis to one of the groups. This would warrant that they both started with the same opportunities for success or beneficial effects.

Participants suffering from a psychiatric condition (for example, binge eaters) will be excluded.

#### Type of interventions

This review will consider public health interventions addressing a reduction in SSB consumption or water increase that had a minimum length of 4 weeks of follow-up (from baseline data collection until the first assessment of outcomes) and that were provided mainly at community settings. Studies taking place at clinical locations (for example, a hospital) will only be reviewed if participants had been allocated into intervention or control groups by random methods.

Interventions addressing artificially sweetened drinks/diet beverages (those flavoured with non-energy-yielding sweeteners such as aspartame, sucralose, saccharin, acesulfame potassium, neotame or stevia) [39] will be included if these were used as alternatives for reducing intakes of SSB, and dietary patterns of consumption were reported. Interventions replacing SSB with 100% fruit juice (for a healthier substitution) will not be included as it may be difficult to determine the real concentration of naturally occurring sugars in such drinks which will bias our results. Interventions targeting an increase in fruit and vegetable juice consumption as the primary outcome will also be excluded.

Trials looking at effects of beverage replacement on hormonal response, appetite and subsequent energy intake will not be included.

#### Type of outcome measures

##### Primary outcomes

- ▪ Change in SSB consumption (in millilitres/per day)
- ▪ Change in water intake (in millilitres/per day)

##### Secondary outcomes

- ▪ Presence or absence of specific intervention components such as behaviour change techniques (BCTs).
- ▪ Change in knowledge/attitude/beliefs in regard to SSB and water consumption as measured by an existing taxonomy on BCTs.
- ▪ Changes in physical environments and policies.
- ▪ Changes in health inequalities as measured by interactions between socio-demographic characteristics of participants and interventions' effects/outcomes.

### Search methods for identification of studies

#### Search strategy

The PICO framework (acronym for patient/intervention/comparator and outcome) was used as a first tool to identify pertinent terminology for inclusion in the search strategy. Considering the characteristics of this review ‘setting’ was used instead of the ‘comparator’ category. A combination of keywords relating mostly to interventions, settings and outcomes comprised the searching. Medical subheadings (MeSH) and other controlled vocabulary used in indexed journals were considered for the development of the strategy.

The following databases will be used to search for relevant articles published from January 1990 in any language:

- ▪ OVID Medline
- ▪ Cochrane central register of controlled trials (CENTRAL)
- ▪ EMBASE
- ▪ Scopus
- ▪ Web of Science
- ▪ The Global Health Library
- ▪ Database of Abstracts of Reviews of Effects (DARE)
- ▪ Clinicaltrials.gov
- ▪ The Trials Register of Promoting Health Interventions (TRoPHI)
- ▪ International Clinical Trials Registry Platform (ICTRP)
- ▪ metaRegister of Controlled Trials (mRCT)

Reference lists will also be scanned in order to include missing relevant papers. Selected articles will be imported to an EndNote library. An example of the searching strategy designed and executed in Medline (OVID) can be seen elsewhere [see Additional file 1].

### Data collection and analysis

#### Selection of studies

Two trained reviewers will independently perform an initial screening based on title and abstract. Any disagreements found at this stage will be discussed by them and - if required - resolved by discussion and consultation with a third review author.

A copy of full articles will be obtained for all potentially relevant studies. For unavailable articles at the University of Leeds, authors will be contacted electronically, and papers will be also ordered from the British Library. Any discrepancies that could arise at this stage will also be resolved by consulting a third reviewer.

Agreement between responses will be verified through Cohen’s kappa index of inter-rater reliability. A kappa of 0.7 will be considered as a good level of agreement.

The process of inclusion and exclusion of records at each stage will be guided, documented and described using the preferred reporting items of systematic reviews and meta-analyses (PRISMA) flow chart which is a recognised tool from a group of reviewers, clinicians, editors and consumers seeking to enhance transparency in published systematic reviews [40].

#### Data extraction and management

Data from the studies meeting the inclusion criteria will be entered into Review Manager 5 software in duplicate. Characteristics regarding type of study, allocation concealment, sample size, intervention targets, setting, population’s age, country and year of study, length of the intervention, primary and secondary outcomes, statistical measures, results as well as attrition rates will be fully extracted by two members of the team using an adapted spreadsheet form available from the Cochrane Collaboration [41] and then managed with the aforementioned software.

The following characteristics will be summarised and presented in tables from studies meeting inclusion criteria: study details (author, year of publication, trial design, place of study), study objective and aims, study duration, setting of intervention, content, delivery (frequency, duration and intensity of activities), duration of intervention and follow-up, participants’ characteristics (mean age, sex and other socio-demographic features available), outcome definition and overall main results on primary outcomes.

Authors will be contacted if no definition or description of serving sizes is available within the information of a study. If no response is provided, then a standardised portion or serving of SSB will be imputed, that being 8 fluid oz or approximately 240 mL.

When studies have measured intakes of SSB or drinking water at several points across a given intervention, baseline and the longest follow-up measurement will be used for analysis. If this was not the case, then baseline and post-intervention measurements will be considered. Frequency of consumption of SSBs or water will be analysed and transformed - if necessary - into ‘times per day’. For studies reporting more than one group or category of SSBs without the total, in the first instance the authors will be contacted to determine whether results for total intake are available. If this is not possible then most important type of SSB will be entered into the analysis used in the meta-analysis. This will be determined by agreement within the review team.

Additional information-when available-on equity will be analysed using the PROGRESS framework (which stands for place of residence, race or ethnicity, occupation, gender, religion, education, socioeconomic status, social status) to identify if the intervention had more positive effects in certain participants or groups.

As one of the main objectives of this review is to identify the behaviour change techniques that explain intervention effectiveness, two independent reviewers will judge and code these (both in the intervention and control groups) with help of an existing reliable taxonomy of 26 techniques that has characterised the content of interventions addressing healthy eating amongst obese populations [42]. Description and examples of techniques can be found in the Additional file 2 [43].

#### Statistical analysis

Random-effects meta-analysis will be carried out to produce a pooled estimate of the difference in millilitres (mL) of SSB and drinking water between the intervention and control arms in the studies included in the review. The data will be displayed in forest plots, firstly of all studies in the review and secondly in subgroups according to the following age stages of childhood development and adulthood: 3 to 5 years old, 6 to 12 years old, 13 to 18 years old and 19 and above [5]. If the whole family was targeted, the intervention will be allocated under the latter subgroup. The *I*^2^ test will be used to check for heterogeneity across studies. Results of heterogeneity denoted by *I*^2^ between 25 and 50% will be indicative of moderate heterogeneity, from 50 to 75% of substantial and above 75% of considerable heterogeneity, respectively [41]. If there are sufficient studies available - more than ten studies [41] - a meta-regression will be conducted to explore whether heterogeneity is explained by the BCTs used in the intervention. This will determine whether the use of certain techniques is associated with more effective interventions. Potential confounders will also be taken into account in the analysis such as age, gender, setting of intervention and randomisation. Mean differences and 95% confidence intervals will be used in the analysis of the primary outcome (change in mL of SSBs or water intakes). Reported means together with standard errors will be used to determine this. Odds ratios and 95% confidence intervals will be used for pooling binary outcomes.

Cochrane’s tool of risk of bias will be used to assess quality of studies (both in randomised, non-randomised and cluster-randomised controlled trials) with regard to allocation concealment, sequence generation, blinding, treatment of completers *versus* non-completers, selective reporting and other biases [44]. Cluster RCTs will be assessed as low risk of bias if the unit of analysis was considered at the same level as the allocation (either by school, classes or community), and allocation was carried out on all entities before the intervention had started. In the case of studies that had not accounted for this, then effect estimates and their standard errors from correct analyses of cluster-randomised trials may be meta-analysed using the generic inverse-variance method in RevMan. In addition, intra-cluster correlation coefficients (ICC) will be used to assess variability between and within clusters. Reporting bias will be identified in studies that included outcomes throughout the methodology but were not presented in the results section or referenced in other peer reviewed publications.

Quantitative synthesis will be the desired approach, yet if very small numbers of studies were available and do not allow this or if heterogeneity was found to be too high or unexplained, then a narrative synthesis will be performed instead or the forest plot will be presented without the pooled estimate provided.

## Discussion

The magnitude of the obesity epidemic in both children and adults worldwide urgently demands action and better approaches. Both observational and experimental evidence have successfully demonstrated a link between SSB intake, weight gain and its related comorbidities (that mainly being obesity, metabolic syndrome, CVD and type 2 diabetes). As a result, research has suggested that SSB are a feasible target for public health initiatives in order to reduce the obesity prevalence and other negative consequences [9,28].

There has been a growing debate in regard to a causative link between sugar-sweetened beverages and weight gain in recent years [9,35,45]. Nevertheless, much of the attention given has focused solely on the longer health outcomes related to morbidity rather than those related to behaviour change. The former could be the result of the multi-factorial context in which health-related behaviours lie, which pose one of the greatest challenges when seeking to tackle unhealthy dietary patterns [46].

Despite the fact that the complexity of a problem like obesity does not rely on the reduced consumption of a single food item (in this case, sugar-sweetened beverages), it should be recognised that their nutritional composition is poor as they do not provide any real health benefit nor appear to have protective effects in any published study so far. Thus, considering the documented parallel increase in consumption trends and obesity rates in many countries, there is much expectation to know the feasibility of generating a change in SSB intake and whether current resources should be kept or placed elsewhere.

The findings derived from this systematic review and meta-analysis will therefore help in the development of improved public health initiatives tackling obesity, particularly in countries with a magnified consumption of SSB. It will also help identify the pathways and discriminate amongst the array of possibilities available to generate a desirable and sustainable change towards healthier drinking patterns.

While it is likely that the number of papers available addressing the intended research questions will be limited - as it is a topic that has gained recent momentum - this review will particularly be benefited by including literature in any language. Consequently, it will be possible to detect useful, innovative strategies or elements that could be integrated in upcoming interventions or programmes for future research and policy making.

## Additional files

Additional file 1:
**Example searching strategy: Medline (Ovid).** This document displays an example of a searching strategy designed and executed in Medline (OVID). Additional file 2:
**Behaviour change techniques used in interventions targeting healthy eating.** This document shows the list and description of each of the techniques that will be used to characterise the intervention’s components. Certain health examples have been also provided.

## Abbreviations

BCT: behaviour change technique
CVD: cardiovascular disease
FAO: Food Agriculture Organisation
*I*^2^: heterogeneity
mL: millilitres
NGO: non-government organisation
oz: ounce
RCT: randomised controlled trial
SSB: sugar-sweetened beverage
UK: United Kingdom
WHO: World Health Organisation
