# Supplementary material for: Impact of interventions to reduce sugar-sweetened beverage intake in children and adults: a protocol for a systematic review and meta-analysis
Source: Syst Rev. 2015 Feb 21;4:17. doi: 10.1186/s13643-015-0008-4 (PMC4343267; doi:10.1186/s13643-015-0008-4)
Supplement: Additional file 1: — Example searching strategy: Medline (Ovid). This document displays an example of a searching strategy designed and executed in Medline (OVID). [file 13643_2015_8_MOESM1_ESM.pdf]

Additional file 1. Example searching strategy: Medline (Ovid)

**Searching strategy: Medline**	
1	((sugar$ adj2 beverage$) or (sugar$ adj2 drink$) or (carbonated adj beverage$) or (carbonated adj drink$) or (fizzy adj drink$) or (fizzy adj beverage$) or (liquid$ adj carbohydrate$) or cordial$ or (cola adj beverage$) or (cola adj drink$)).tw.
2	("sugar sweetened beverage$" or "sugar-sweetened beverage$").tw.
3	exp Carbonated Beverages/
4	(sugar containing adj (beverage$ or drink$)).tw.
5	(non alcohol$ adj2 (beverage$ or drink$)).tw.
6	("high energy beverage$" or "high energy drink$").tw.
7	(energy adj2 (drink$ or beverage$)).tw.
8	("sweet$ caloric beverage$" or "sweet$ caloric drink$").tw.
9	(sweet$ adj2 (beverage$ or drink$)).tw.
10	((added adj2 sugar$) and (beverage$ or drink$)).tw.
11	(sugar$ rich adj2 (drink$ or beverage$)).tw.
12	(fruit adj2 juice$).tw.
13	1 or 2 or 3 or 4 or 5 or 6 or 7 or 8 or 9 or 10 or 11 or 12
14	((non calori* sweet* and (beverage$ or drink$)) or (non nutriti* sweet* and (beverage$ or drink$))).tw.
15	(reduced calori* adj2 (beverage$ or drink$ or juice$)).tw.
16	((sucralose adj2 (beverage$ or drink$ or juice$)) or (neotame adj2 (beverage$ or drink$ or juice$)) or (acesulfame adj3 (beverage$ or drink$ or juice$)) or (saccharin adj2 (beverage$ or drink$ or juice$))).tw.
17	((diet* adj2 (beverage$ or drink$ or juice$)) or (low calori* adj (beverage$ or drink$ or juice$))).tw.

18	(artificial$ sweet$ adj2 (beverage$ or drink$ or juice$)).tw.
19	(artificial* sweet$ and (beverage$ or drink$ or juice$)).tw.
20	((intense sweetener$ and (beverage$ or drink$)) or (intense sweet$ adj3 (beverage$ or drink$))).tw.
21	((sweetening agent and (beverage$ or drink$)) or (artificial$ sweet$ adj2 (beverage$ or drink$))).tw.
22	(sugar-free adj2 (beverage$ or drink$)).tw.
23	14 or 15 or 16 or 17 or 18 or 19 or 20 or 21 or 22
24	(sport$ adj2 (drink$ or beverage$)).tw.
25	13 or 24
26	(health$ adj2 behavio#r).tw.
27	exp Health Behavior/ or exp Health Education/ or exp Health Promotion/
28	(health education or health promotion).tw.
29	((community adj2 intervention$) or (media adj2 intervention$)).tw.
30	((community or school) adj2 (intervention$ or program$)).tw.
31	((family adj2 intervention$) or (parent$ adj2 intervention$)).tw.
32	((lifestyle or life style) adj2 (chang$ or intervention$)).tw.
33	(behavio#r adj (chang$ or intervention$ or strateg$)).tw.
34	(health$ adj2 (attitude$ or school$)).tw.
35	exp Health Knowledge, Attitudes, Practice/
36	(diet$ adj (chang$ or education or behavio#r or pattern$)).tw.
37	exp health policy/ or exp nutrition policy/
38	((nutrition$ adj3 polic$) or (food adj polic$) or (school$ adj polic$)).tw.
39	26 or 27 or 28 or 29 or 30 or 31 or 32 or 33 or 34 or 35 or 36 or 37 or 38
40	randomi#ed controlled trial.pt.

41	controlled clinical trial.pt.
42	randomi#ed.ab.
43	randomly.ab.
44	trial.ab.
45	groups.ab.
46	exp intervention studies/ or exp pilot projects/ or exp comparative study/
47	40 or 41 or 42 or 43 or 44 or 45 or 46
48	limit 47 to yr=1990-2014
49	exp Drinking Water/
50	(water adj3 provision).tw.
51	(filter$ adj water).tw.
52	(water adj2 (increas$ or intake$ or consum$)).tw.
53	(drinking water adj (increas$ or intake$ or consum$ or decreas$ or reduction$)).tw.
54	(plain adj2 water).tw.
55	49 or 50 or 51 or 52 or 53 or 54
56	exp animals/ not (exp animals/ and exp humans/)
57	exp Veterinary Medicine/
58	exp Animal Experimentation/
59	56 or 57 or 58
60	(25 and 39 and 48) not 59
61	(23 and 39 and 48) not 59
62	(55 and 39 and 48) not 59
