# Supplementary material for: Impact of interventions to reduce sugar-sweetened beverage intake in children and adults: a protocol for a systematic review and meta-analysis
Source: Syst Rev. 2015 Feb 21;4:17. doi: 10.1186/s13643-015-0008-4 (PMC4343267; doi:10.1186/s13643-015-0008-4)
Supplement: Additional file 2: — Behaviour change techniques used in interventions targeting healthy eating. This document shows the list and description of each of the techniques that will be used to characterise the intervention’s components. Certain health examples have been also provided. [file 13643_2015_8_MOESM2_ESM.pdf]

Additional file 2. *Behaviour-change techniques used in interventions targeting healthy eating*

Number of technique	Technique	Description [43]	Health examples
(T1)	Provide information on behaviour–health link.	Information about the relationship between the behaviour and its possible or likely consequences in the general case usually based on epidemiological data and not personalised for the individual or health education material relevant to the behaviour.	Provide general information about consequences of high intakes of SSB consumption (risk of obesity, metabolic syndrome, type 2 diabetes, coronary heart disease).
(T2)	Provide information on consequences to the individual	Information about the benefits and costs of action or inaction to the individual or tailored to a relevant group based on that individual's characteristics (demographics, clinical, behavioural or psychological information).	Provide information to participants about benefits of reducing SSB intake and the costs of high SSB consumption.
(T3)	Provide information about others' approval	Involves information about what other people think about the target person's behaviour. Clarifies whether others will like, approve or disapprove of what the person is doing or will do.	
(T4)	Prompt intention formation	Involves encouraging the person to set a general goal or make a behavioural resolution e.g., "I will take more exercise next week" would count as a prompt to intention formation. This is directed towards encouraging people to decide to change	Encourage participants to make behavioural resolution such as:<br>- "I will drink less SSB this week"
(T5)	Prompt barrier identification	Presumes having formed an initial plan to change behaviour. The person is prompted to think about potential barriers and identify the ways of overcoming them. Barriers may include challenging goals in specified circumstances. Concerns behavioural, cognitive, emotional, environmental, social and/or physical barriers.	Identify obstacles at particular instances (such as school or workplaces) in which desired behaviour may be compromised (i.e vending machines with unhealthy drinks) and seek potential solutions to face them (i.e increase water intake by bringing and re-filling a reusable bottle every day).
(T6)	Provide general encouragement	Involves praising or rewarding the person for effort or performance without making this contingent on specific behavioural performance; or "motivating" the person in an unspecified manner. This will include attempts to enhance self-efficacy through argument or persuasion (e.g., telling someone the will be able to perform a behaviour).	

Number of technique	Technique	Description [43]	Health examples
(T7)	Set graded tasks:	Breaking down the target behaviour into smaller easier to achieve tasks enabling the person to build on small successes to achieve target behaviour.	Breaking down target behaviour related to SSB or water intake such as:<br>- Get a re-usable bottle to drink more water.<br>- Remember to bring bottle at school/workplace and refill it with water.
(T8)	Provide instruction on how to perform the behaviour:	Involves telling the person how to perform behaviour or preparatory behaviours, either verbally or in written form. Cooking and exercise classes as well as personal trainers and recipes should always be coded as this technique or T9.	
(T9)	Model/demonstrate the behaviour	Involves showing the person how to perform a behaviour e.g through physical or visual demonstrations of behavioural performance, in person or remotely. Participant "observes" behaviour being enacted.	Demonstrate desirable behaviour by showing participants, for example, how to make a healthier drink.
(T10)	Prompt specific goal setting (behaviour)	The person is encouraged to make a behavioural resolution (take more exercise during the week). Encouraging people to decide to change or maintain change. Different from goal setting outcome as It does not involve planning exactly how the behaviour will be done and either when or where he behaviour or action sequence will be performed.	Motivate participants to make resolution on desired/targeted behaviour. Example:<br>- If thirsty, I will only drink plain water instead of soft drinks.
(T11)	Prompt review of behavioural goals	Involves a review or analysis of the extent to which previously set behavioural goals were achieved (i.e take more exercise next week). Follows setting goals' technique and persons' revision/readjustment to achieve them.	
**Number of technique**	**Technique**	**Description [43]**	**Health examples**
(T12)	Prompt self-monitoring of behaviour	The person is asked to keep a record of specified behaviours as a method for changing behaviour. Should be completely stated as *intervention component* (diary, completing questionnaire on physical activity).	Use of beverage logs to keep track of intake of liquids/fluids throughout the day.
(T13)	Provide feedback on performance	Involves providing the participant with data about their own recorded behaviour or commenting on a person's behavioural performance –or a discrepancy between one's own performance in relation to others.	

(T14)	Provide contingent rewards	Involves the person using praise or rewards for attempts at achieving a behavioural goal. Might include efforts made towards achieving the behaviour or progress made in preparatory steps towards the behaviour, but not merely participation in intervention. This can include self-reward.	Consists of rewarding attempts for behavioural change or improvements achieved. Example:<br>- Entering participants seen drinking water in a drawing for prizes.
(T15)	Teach to use prompts/cues	The person is taught to identify environmental prompts which can be used to remind them to perform the behaviour (or to perform an alternative, incompatible behaviour) in the case of behaviours to be reduced. Cues could include times of day, particular contexts or technologies such as mobile phone alerts which prompt them to perform the target behaviour.	Identification of cues to engage in desired behaviour such as media messages discouraging intake of SSB.
(T16)	Agree a behavioural contract	Involves written agreement on the performance of an explicitly specified behaviour so that there is written record of the person's resolution witnessed by another.	
(T17)	Prompt practice	Prompt the person to rehearse and repeat the behaviour or preparatory behaviours numerous times. Described as "*building habits or routines*" but is still practice so long as the person is prompted to try the behaviour during the intervention or practice between intervention sessions (i.e as homework).	
(T18)	Use of follow-up prompts	Intervention components are gradually reduced in intensity, duration and frequency over time (e.g telephone or letters instead of face to face sessions and/or provided at longer time intervals).	Use of text messaging.

**Number of technique**	**Technique**	**Description**	**Health examples**
(T19)	Provide opportunities for social comparison	Involves explicitly drawing attention to other's performance to elicit comparisons.	Necessarily involves a comparison of how an individual's performance compares to others- as it relates to opportunities. Any group-based approach is coded yes for this technique as it provides an opportunity *per se* for social comparison.
(T20)	Plan social support/social change	Involves prompting the person to plan how to elicit social support from other people to help him/her achieve their target behaviour/ outcome. Includes support during intervention (i.e. *buddy system*) and at	

		follow up, support provided by the individuals delivering the intervention, partner, friends and family (supporting systems).	
(T21)	Prompt identification as role model/ position advocate	Involves focusing on how the person may be an example to others and affect their behaviour. Also includes opportunities for participants to persuade others of the importance of adopting/changing the behaviour.	Stress role of participant in others' behaviour (i.e parents' intake of SSB and consequently their children's intake).
(T22)	Prompt self-talk	Encourage the person to use talk to themselves (Aloud or silently) before and during planned behaviours to encourage, support and maintain action.	
(T23)	Relapse prevention	Identify situations that increase the likelihood of the behaviour not being performed and apply coping strategies to those situations.	
(T24)	Stress management	Behaviours undertaken to reduce stressors or impact of stressors.	
(T25)	Motivational interviewing	Elicit self-motivating statements and evaluation of own behaviour to reduce resistance to change.	
(T26)	Time management	Action planning applied to the perceived problem of shortage of time.	
